# Characteristics and Material Flows of Diary Packaging in Municipal Solid Waste: A Case Study of Plastic Yoghurt Cups in Vienna

**DOI:** 10.1002/gch2.70144

**Published:** 2026-08-12

**Authors:** Gisela Breslmayer, Lea Gritsch, Jakob Lederer

**Affiliations:** ^1^ CD Laboratory for a Recycling‐Based Circular Economy Institute for Chemical Environmental and Bioscience Engineering Technische Universität Wien Wien Austria

**Keywords:** consumer behavior, design‐for‐recycling, manual sorting analysis, material flow analysis, packaging waste, sustainability, yoghurt cups

## Abstract

Yoghurt cup packaging has received increasing attention regarding design‐for‐recycling. Packaging technologies like the three‐component (3C) cup, consisting of a plastic cup, carton wrap and aluminum cover, reduce the amount of plastic needed. While individual components are well recyclable if consumers correctly separate and dispose of them after consumption, no data regarding consumer separation behavior of yoghurt cups is available. This study offers a detailed characterization of 3C cups and other yoghurt cup decoration types (2C) regarding their dismantling stage and content in mixed municipal solid waste and lightweight packaging waste in Vienna, Austria. The results show that 3C cups represent one of the largest shares of yoghurt cup decoration types, totaling over 42 Mio cups/year (645 t/year) in Vienna. Both 2C and 3C cups are better dismantled when separately collected. However, 95% of all cups land in mixed municipal solid waste and are unavailable for recycling. Separately collected 3C cups with carton wrap additionally hinder the correct sorting of cups in a sorting facility. Lacking participation of consumers regarding dismantling and correctly disposing of yoghurt cups can therefore compromise design‐for‐recycling efforts. Continued monitoring of yoghurt cup quality and content in different waste streams is recommended to better understand consumer behavior.

## Graphical abstract

Consumer dismantling behavior of yoghurt cups has been quantified in Vienna. Three‐component cups (with carton wrap) are increasingly present on the market but are rarely correctly dismantled and disposed of correctly, leading to mis‐sorting and loss of recyclable material. With 95% of yoghurt cups being disposed of in mixed municipal solid waste, the need for improved consumer participation is highlighted.

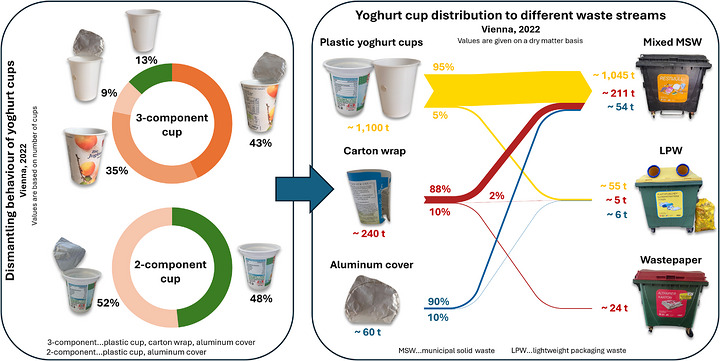

## 1 Introduction

Plastic demand and production have been increasing globally since the 1940s due to the material's cost‐effectiveness and customizability [1], with 387 Mt of fossil‐based plastics produced worldwide in 2024 [2]. In Europe, 39% of plastic is used for packaging, mainly for food and beverages. While plastic packaging protects goods and can minimize food waste [3, 4], short use phases contribute to increased plastic packaging waste [5], amounting to 35 kg per capita in the EU in 2023 [6]. This presents a challenge for waste management with consequences for human health, the environment, and the economy [7, 8].

Shifting from a linear toward a circular economy of plastic packaging is therefore one of the great intended transformations of contemporary societies to overcome these challenges [9, 10]. Actors involved are aiming to achieve a circular economy of plastic packaging by fostering measures such as the reduction of consumed amounts of raw material, as well as improvements in the recycling of waste [11]. The latter can be positively influenced by design‐for‐recycling to create recyclable products and ensure their usage as secondary raw material at the end‐of‐life [12, 13, 14, 15]. Despite these options, achieved recycling rates of plastic packaging are still much lower than those for glass, metals, or paper packaging, leading to a low supply of secondary raw materials available and low recycled material contents in plastic packaging [16, 17, 18, 19]. Targets like a global plastics recycling rate of 42% by 2040 [20] or the EU's Packaging and Packaging Waste Regulation (PPWR), which mandates the recyclability of all packaging by 2030 [21], therefore require large efforts from the plastic packaging industry to implement design‐for‐recycling [22, 23, 24, 25]. Currently, plastic packaging is very heterogeneous regarding used packaging and polymer types or decoration technologies [26, 27, 28], while design‐for‐recycling aims for simplified packaging, for example by avoiding multilayer design or opaque coloring [29]. In addition to packaging design, correct separation and disposal of packaging by consumers are fundamental for achieving recycling targets. These are influenced by multiple factors such as the collection scheme, the perceived packaging value, or demographic factors [30, 31, 32].

A product that has recently attracted increased attention in literature with respect to design‐for‐recycling is yoghurt packaging. Focus has been put on these cups especially regarding their emptiability and their usage for recycled polymer pellets [33, 34, 35, 36]. While various packaging technologies are used for yoghurt [37, 38], in many countries it is primarily packaged in two‐component (2C) yoghurt cups made of polypropylene (PP) or polystyrene (PS) and sealed with an aluminum cover [39, 40, 41, 42, 43, 44]. Recently, an increasing trend of carton‐wrapped plastic cups can be observed, especially in the DACH region (Germany, Austria, Switzerland) [35, 41, 42], where a total of 2 Mt of yoghurt were produced in 2024, with Germany being the largest yoghurt producer in the EU‐27 [45, 46]. This type of yoghurt cup, as shown in Figure 2, consists of 3 components (3C) (plastic cup + carton wrap + aluminum cover) and offers the advantage of less plastic being needed for the stabilization of the cup [42]. The carton wrap itself acts as an additional layer of protection for the contained product and enables communication with the consumers through printing [47]. This eliminates the need for further decoration technologies on the plastic cup itself, thereby positively influencing the cup's recycling properties [48]. Schlossnikl et al. [49], for instance, showed that 3C cups show high homogeneity in recycling regarding their mechanical properties and color when compared to 2C cups with direct print. Whilst contaminants, such as residues of adhesives or paper fibers, can remain on the plastic cup after separation (see Figure 2), their removal is possible during industrial reprocessing by wind sifting or friction washing [50]. Additionally, paper‐based packaging is positively perceived by consumers regarding its recyclability and eco‐friendliness [31, 51, 52], a perception supported by high carton recycling rates in many countries [18, 53, 54]. Furthermore, the recycling of the aluminum cover can substantially reduce greenhouse gas emissions if compared to aluminum produced from bauxite [55]. The high recyclability of the 3C cups subcomponents is, however, dependent on the dismantling behavior of consumers after consumption and the correct disposal of each component into the respective separate collection scheme. Separation of the cup's subcomponents after consumption, especially of the carton wrap, is often encouraged on the packaging itself (see Figure S1). The consumers' participation, however, is questioned by Schroeder [56], while Klein et al. [41] state that in this regard, “no empirical studies on actual consumer behavior are available”. Furthermore, Gürlich et al. [48] point out that separation of packaging components should not depend on the consumers, as it is difficult to influence their behavior. While the German “Mindeststandard” shares this view and thus classifies 3C cups as 0% recyclable, Austrian standards rate their recyclability at 96% [41]. The new PPWR [21] does not classify 3C cups as composite packaging since a manual separation is possible; design‐for‐recycling guidelines, however, do not recommend paper‐based materials in combination with plastics, as it hinders correct polymer recognition during the sorting process and lowers the quality of the recycled polymer if not dismantled and disposed of correctly [57, 58].

This partly controversial discussion calls for data on the actual market size and the dismantling and separation behavior of consumers regarding 3C and 2C yoghurt cups. According to Köck et al. [59], publicly available market surveys are only available at the micro level, like the study from Kostic et al. [42], who analyzed decoration technologies for yoghurt cups found in supermarkets in Munich, Germany, with 18% being 3C. Köck et al. [59] found a share of 50% 3C cups out of 130 analyzed cups in non‐randomly selected supermarkets in Vienna. Instead of supermarket surveys, however, it is also possible to determine the market size by a combination of waste sampling and analysis, together with material flow modelling, as yoghurt cups usually get disposed of after consumption of the product. This approach further enables the determination of the content and constitution of yoghurt cups at disposal (dismantling stage) both in mixed municipal solid waste (MSW) and lightweight packaging waste (LPW), indicating the dismantling and separate collection behavior of consumers. Approaches like these were used by Gritsch et al. [26] for non‐beverage plastic packaging and by Gritsch et al. [53] for paper‐based packaging in Vienna, Austria, but not for yoghurt cups. Other studies analyzed selected LPW streams but not mixed MSW, like Traxler et al. [35], or investigated very small areas like Köck et al. [59], who present data on yoghurt cups for a negligibly small neighborhood of 10 000 inhabitants in Vienna, a city of 1.93 million. Moreover, none of the studies on yoghurt cups in waste used their data for material flow modelling, even though it not only provides information on market size but also on the dismantling and separate collection behavior of consumers.

This study aims to fill the elaborated research gaps on the market size, contents, constitution, and material flows of yoghurt plastic packaging with carton wrap in comparison to those without carton wrap, using the case study of Vienna. In detail, the following questions are asked:

1. What are the quantities and relative shares of yoghurt plastic packaging cups with and without carton wrap found in mixed MSW for disposal and separately collected lightweight packaging waste?
2. What is the constitution of yoghurt plastic packaging cups with and without carton wrap in the analyzed waste streams, including residues and dirt content, and the extent to which consumers dismantle individual components such as carton wraps and aluminum covers?
3. What are the Material Flows of Yoghurt Plastic Packaging Cups with and Without Carton Wrap and Their Respective Subcomponents Across the Analyzed Waste Streams?

## 2 Materials and Methods

**FIGURE 1:**
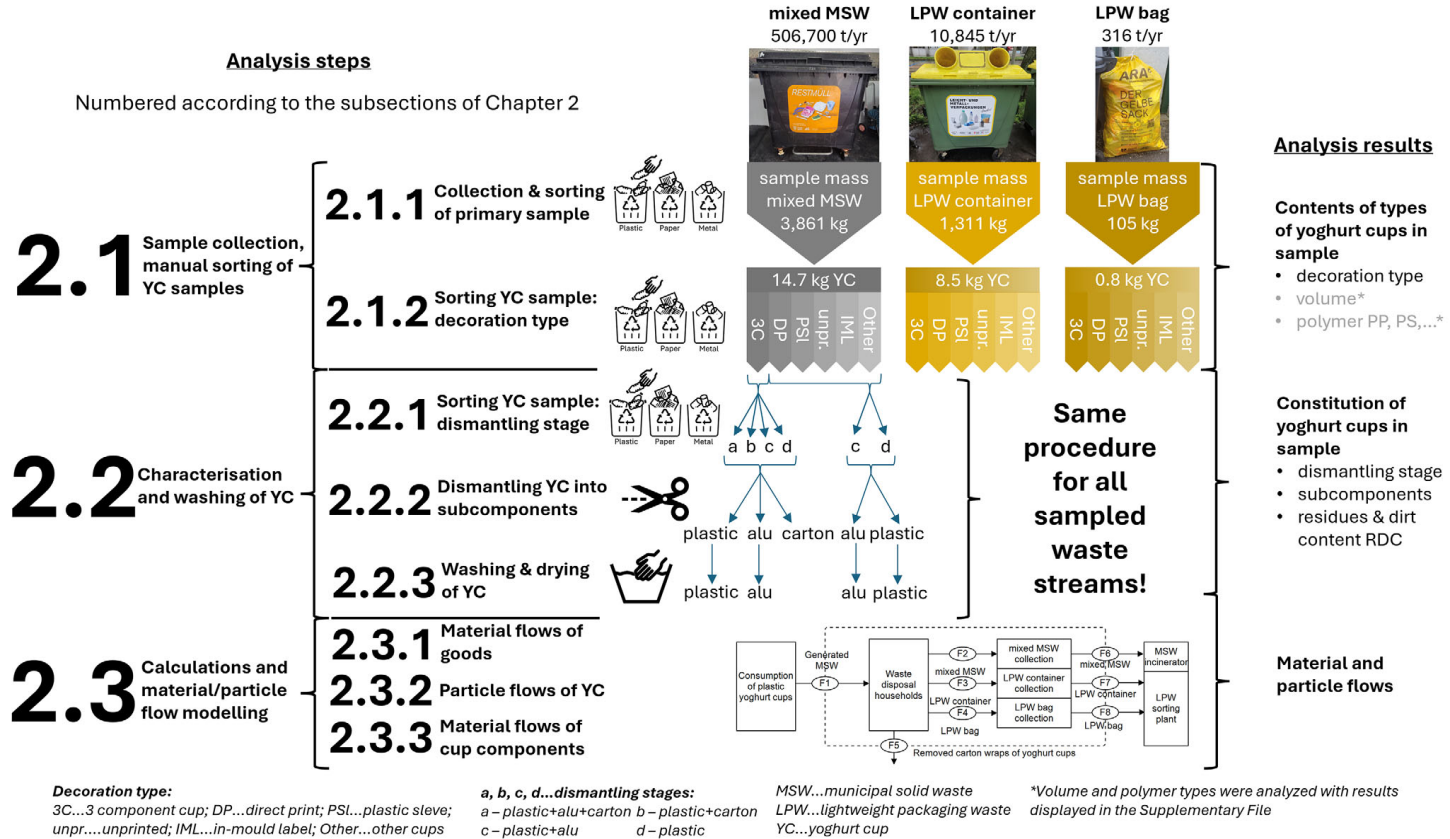
Methodological workflow of the collection, sorting, characterization, washing and calculation of material and particle flows of yoghurt cups. The numbering follows the subsection structure of Chapter 2, indicating where analysis steps are introduced and elaborated in detail.

Vienna, the capital of Austria, was selected as a case study area for two reasons: First, with a population of 1.93 million in 2022 and 1.1 Mt/yr of MSW generated, the city represents 22% of Austria's inhabitants and 16% of the country's yearly MSW generation [60, 61]. Second, the public waste management provider of Vienna, municipal department MA 48, provided access to collected waste samples [62]. Figure 1 presents a graphical summary of the methodological workflow applied in this study. The analytical steps in this figure are numbered according to the subsections in Chapter 2 of this work.

### 2.1 Waste Sampling and Analysis of the Yoghurt Cup Content and Composition

#### 2.1.1 Collection and Analysis of Primary Waste Samples to Determine the Yoghurt Cup Content

Every five to six years, MA 48 conducts an in‐depth waste sampling campaign of all collected MSW streams like mixed MSW and separately collected waste. Samples analyzed in this study originated from the latest campaign in 2022, which was conducted in accordance with Austrian guidelines [63]. Based on past waste sampling analyses, it was known that only three waste streams contain yoghurt cups: mixed MSW, LPW collected in containers, and LPW collected in bags [62, 64, 65]. Thus, only these waste streams were analyzed in detail for the presence of yoghurt cups. Over the 15‐day sampling period, 3681 kg of mixed MSW, 1311 kg of LPW from containers, and 105 kg of LPW from bags were randomly collected, manually sorted, and analyzed together with an engineering company. For a detailed description of the sampling campaign, see Gritsch et al. [26].

#### 2.1.2 Sorting of Yoghurt Cups According to Their Decoration Type

The total amount of yoghurt cups found in all three waste streams during the sampling campaign (14.7 kg in mixed MSW, 8.5 kg in LPW containers and 0.8 kg in LPW bags, based on wet matter basis) were further sorted based on production and decoration type, distinguishing between 3C cups consisting of three material components (plastic cup, carton wrap, aluminum cover) and cups of other decoration types, namely direct‐print (DP), plastic sleeve (PSl), unprinted (unpr.), in‐mould labelling (IML), and other decoration types. All of these were summarized as 2C cups since they consist of two components (plastic cup, aluminum cover). Exemplary pictures of the listed cup decorations can be found in Figure S2. Other properties such as the filling volume (≤250 mL and >250 mL) and the polymer type (PP, PS, PET) were analyzed as well but are only displayed in Table S1 and Figure S5, due to not being the focus of this article. For the analysis, the yoghurt cups in the samples were first sorted and then counted to determine the particle (*p*) and mass (*m*) content after Equations (1) and (2).

(1) $$c_{yip,S}=n_{yip,S}\;/m_{i,S}$$

(2) $$c_{yim,S}=m_{yi,S}\;/m_{i,S}$$

*c*_*yip*,*S*_ is the particle (*p*) and *c*_*yim*,*S*_ the mass (*m*) content of the different decoration types *y* (e.g., 3C cup, DP cups, etc.) in the sample *S* of material flow *i*, which is the analyzed waste stream (mixed MSW, LPW container, LPW bag). *n*_*yip*,*S*_ is the number of particles of a certain subgood category *y* in the sample *S* of material flow *i*, while the total sample mass of each sampled material flow *i* is *m*_*i*,*S*_. *m*_*yi*,*S*_ means the same as *n*_*yip*,*S*_ but by mass of all particles of subgood *y*.

### 2.2 Constitution, Subcomponents, Residues and Dirt Content of Yoghurt Cups in Samples

#### 2.2.1 Constitution (Dismantling Stage)

**FIGURE 2:**
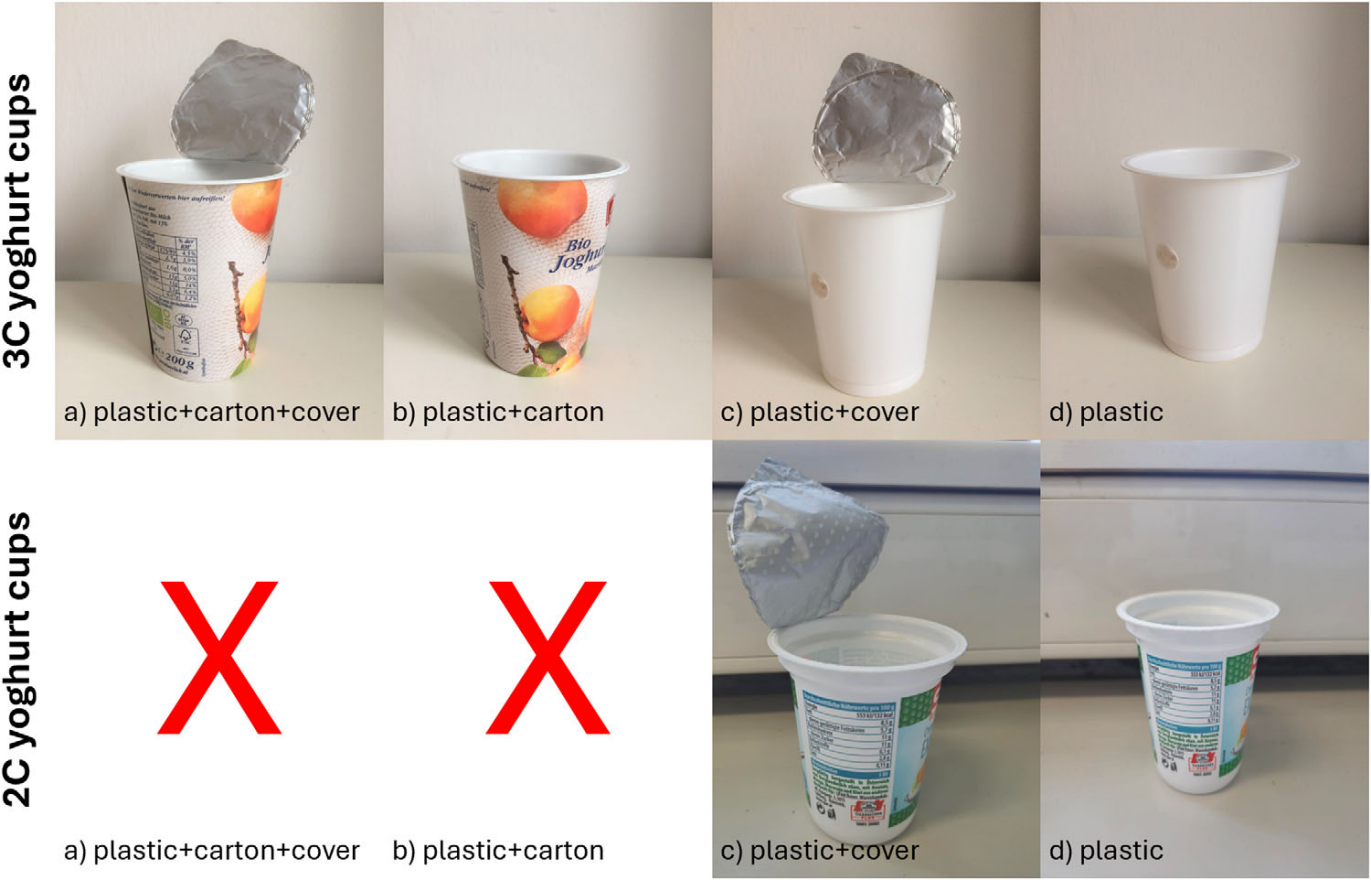
Different dismantling stages of a disposed 3‐component (3C) cup in comparison to 2‐component (2C) cups. Pictures by Lea Gritsch and Gisela Breslmayer.

The term constitution expresses the dismantling stage at which consumers dispose of yoghurt cups. The dismantling stage refers to the degree of separation of an analyzed yoghurt cup. Cups of different decoration types were sorted according to their dismantling stage‐based constitution. 3C cups were distinguished as: a) complete cup (plastic cup + carton wrap + aluminum cover); b) removed cover (plastic cup + carton wrap); c) removed carton wrap (plastic cup + aluminum cover); d) all subcomponents removed (plastic cup). 2C cups were distinguished in these with and without aluminum cover (dismantling stages c and d), as shown in Figure 2 below. After sorting, the wet mass *m* of the 3C cups and the different 2C cup types *y* for the sample *S* of each sampled waste stream *i*, namely *m*_*yi*,*S*_, was determined.

#### 2.2.2 Analysis of Subcomponents

For 3C cups, the subcomponents are the plastic cup, the carton wrap, and the aluminum cover; for 2C cups, the plastic cup and the aluminum cover. Due to prior dismantling by consumers, not all subcomponents were present in the sampled yoghurt cups. Nevertheless, all cups were completely dismantled according to these subcomponents, distinguishing between decoration types *y* on the one hand, and dismantling stage on the other hand. Then, the subcomponents were weighed to determine their wet mass *m* for each decoration type *y* for the sample *S* of each sampled waste stream *i*. The result is *m*_*yi*,*S*, *subcomponent*_. By dividing *m*_*yi*,*S*, *subcomponent*_ by the mass of the sample and each waste stream, *m*_*i*,*S*_, the mass contents of the subcomponent in the sample of each waste stream *c*_*yi*, *subcomponent*_ were calculated (see Equation 3).

(3) $$c_{yi,S,\;subcomponent}=m_{yi,S,subcomponent}\;/m_{i,S}$$

#### 2.2.3 Residues and Dirt Content (RDC)

Weighing the cups before and after washing and drying enables the determination of the RDC content for each subgood *y* in the sample *S* of the sampled waste stream *i* (*RDC*_*yi*,  *S*_). Gritsch et al. [26] and Thoden van Velzen et al. [66] determined the RDC according to Equation (4), where *m*_*gross, iy*_ represents the mass of decoration type *y* in the sample *S* of waste stream *i* on a wet matter basis while *m*_*net, iy*_ is the mass of subgood *y* in waste stream *i* after washing and drying.

(4) $$\begin{array}{ccc} RDC_{yi,S} & & =\frac{m_{yi,S,gross}-m_{yi,S,net}}{m_{yi,S,gross}}\\ & & =\frac{\sum m_{yi,S,subcomponent,gross}-\sum m_{yi,S,subcomponent,net}}{\sum m_{yi,S,subcomponent,gross}}\; \end{array}$$

### 2.3 Modelling Material and Particle Flows of Yoghurt Cups in Vienna

**FIGURE 3:**
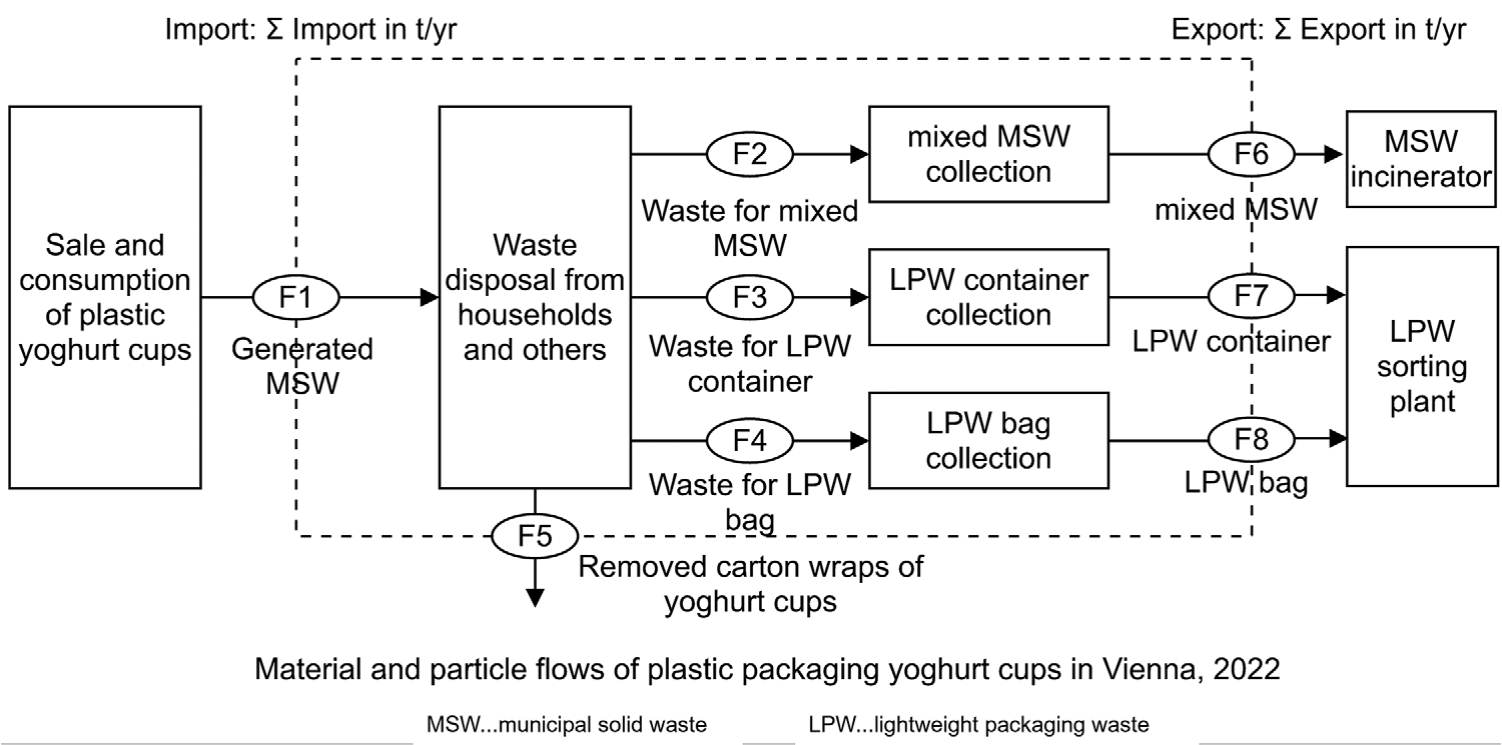
Material flow analysis (MFA) and material‐particle flow analysis (MPFA) model of plastic yoghurt cups in Vienna, 2022.

Material flows of yoghurt packaging and their subcomponents were modelled using a combined material‐particle flow analysis (MPFA) (see Section 2.3.2), based on material flow analysis (MFA) [67]. The geographical system boundary for this MPFA is Vienna, with the year 2022 being the temporal boundary, as shown in the MFA/MPFA‐model in Figure 3.

Processes in the system encompass waste disposal (by households and other MSW generators) and the collection of mixed MSW, LPW containers, and LPW bags. Processes of production and consumption, as well as incineration of mixed MSW and treatment of LPW, are outside of this system's boundary, as well as littering. A recent study on littering in Austria did not report yoghurt cups as a distinct fraction but subsumed them under general food packaging, suggesting that yoghurt cups are not a major contributor to littering [68]. Collected waste flows by MA 48, Vienna's public waste operator, containing yoghurt were chosen as considered material flows for this model. These are: generated MSW (F1), disposed of waste for mixed MSW (F2), waste for LPW container (F3), waste for LPW bag (F4), collected mixed MSW (F6), LPW container (F7), and LPW bag (F8). Since there is no change in the mass during collection, the following relation between material flows applies: F2 = F6, F3 = F7, and F4 = F8. In addition, a waste flow specifically for removed carton wraps of 3C cups was introduced to represent their disposal to other waste streams, particularly to separate wastepaper collection (F5) (see Section 2.3.3). Figure S3 shows the different collection schemes. Mixed MSW is collected by MA 48 directly from households at least on a weekly basis and comprises all waste that cannot be further recycled, except bulky waste. LPW, however, is either collected in containers situated at collection points or directly from households in yellow bags. The latter collection option is limited to single‐family households of selected districts in Vienna [16, 69, 70]. At the time of the study, targeted fractions for LPW collection encompassed plastic bottles, beverage cartons and metals, as is further elaborated in Chapter 3.5. A more detailed description of waste management in Vienna is provided in Gritsch and Lederer [16].

#### 2.3.1 Material Flows of Goods

Material flows containing yoghurt cups were defined as goods, specifically waste for mixed MSW, LPW container and LPW bag collection. By using Equation (5) and data provided by MA 48 [62], these material flows of goods were calculated. In detail, ${\dot{m}}_{input}$ is the material flow of goods entering a process or system, in this case generated MSW, and $\sum_{i=1}^{i=l}{\dot{m}}_{i,output}$ is the sum of *l* output material flows *i* leaving the process or system. The output material flows *i*, *output* for Vienna, 2022 were mixed MSW (506 700 t/yr), LPW container (10 845 t/yr), and LPW bag (316 t/yr). Mass percentage shares of yoghurt cups in all analyzed waste streams are presented in Chapter 3.1; mass information in Chapter 3.4.

(5) $${\dot{m}}_{input}=\sum_{i=1}^{i=l}{\dot{m}}_{i,output}\;$$

#### 2.3.2 Particle Flows of Subgoods

Goods were further divided into subgoods [67], namely different types of yoghurt cups according to their decoration type *y*. Therein, decoration type 3C was distinguished from 2C without carton wrap. Contrary to standard MFAs, the material flows of subgoods in this study are expressed and calculated not in mass flows (i.e., mass unit per time unit), but in particle flows, meaning in number of particles per time unit, for the following reason: while some yoghurt cups may have been identical (same product and packaging components) before entering the waste system, the mass of these particles may differ due to dismantling by the consumers or subcomponents getting lost in the collection vehicle. Using particles as a reference unit enabled the calculation of the total amount of yoghurt cups in F1, which was also a requirement to calculate the material flows of subcomponents (see Section 2.3.3).

While the particle flow calculation of the different yoghurt cup types in material flows F2, F3, F4, F6, F7, and F8 used the results from the sampling campaign (see Section 2.1.2), the particle flow in F1 was calculated using Equation (6) with ${\dot{n}}_{p,input,y}$ being the total number of cup particles for each subgood (or decoration type) *y* entering the system. This corresponds to the market share of each of these yoghurt cup types. To determine this value, material flows ${\dot{m}}_{output,yi}$ of the output (in t/yr) were multiplied by the determined particle contents *c*_*p, output*, *yi*_ in *i*  =  *l* output material flows. These particle contents *c*_*p, output*, *yi*_ were assumed to be equal to the ones determined for the sampled output flows (*c*_*yip, S*_ as determined in Equation 1).

(6) $${\dot{n}}_{yp,input}=\sum_{i=1}^{i=l}{\dot{m}}_{yi,output}\times c_{yip,output}\;$$

By building the sum over all subgoods or decoration types *y*, the total number of yoghurt cups in Vienna's waste streams was determined by applying Equation (7). Therein, ${\dot{n}}_{yp,input}$ was determined after Equation (6), and the sum was built over *y*  =  *k* subgoods, or decoration types.

(7) $${\dot{n}}_{p,input}=\sum_{y=1}^{y=k}{\dot{n}}_{yp,input}\;$$

#### 2.3.3 Material Flows of Subcomponents

Lastly, material flows of yoghurt cup subcomponents were modelled. As mentioned in Section 2.3.2, yoghurt cups found in the analyzed waste streams were partly missing subcomponents. To determine the mass of all subcomponents in flow F1, data of yoghurt cups containing all these subcomponents were used (see Section 2.2.3). Equation (8) shows the calculation of the mass for each subcomponent entering the system. The mass of non‐removed subcomponents from each decoration type is *m*_*yp, subcomponent*_, with ${\dot{n}}_{yp,input}$ being the particle content of each decoration type, the latter derived from Equation (6).

(8) $${\dot{m}}_{y,subcomponent,input}=m_{y\;p,subcomponent}\;\times {\dot{n}}_{yp,input}$$

To model the material flows of the subcomponents in F2 = F6 (mixed MSW), F3 = F7 (LPW container), and F4 = F8 (LPW bag), their content in each of these flows, as determined in Equation (3) (*c*_*yi*,*S*,  *subcomponent*_) was multiplied by the regarding material flow of goods ${\dot{m}}_{i}$, following Equation (9). The data for ${\dot{m}}_{i}$ is shown in Section 2.3.1.

(9) $${\dot{m}}_{yi,subcomponent}={\dot{m}}_{i}\;\times c_{yi,\;subcomponent}$$

While the data for the subcomponent plastic cup was available for all flows based on the prior calculations and sampling, assumptions had to be made for carton wrap and aluminum cover subcomponents that have been dismantled by customers but were not found in the sample. Therefore, the transfer function of MFA was used as shown in Equation (10).

(10) $${\dot{m}}_{yi,subcomponent}={\dot{m}}_{y,subcomponent,input}\;\times TC_{yi,\;subcomponent}$$

Therein, ${\dot{m}}_{yi,subcomponent}$ is the mass of the regarding subcomponent in decoration type *y* and material flow *i*, calculated by the mass of the subcomponent of the subgood in the input ${\dot{m}}_{y,subcomponent,input}$ multiplied by the transfer coefficient *TC*_*yi*, *subcomponent*_ of this subcomponent *y* to an output material flow *i*. To apply Equation (10) to determine the carton wraps disposed of to other waste streams, particularly wastepaper collection (F5), the separate collection rate of 45% for this packaging type of Gritsch et al. [53] was used, leading to a *TC*_*y, waste* *paper* *collection*,  *carton*_ =  0.45. The remainder is assumed to be disposed of via mixed MSW (F2), since it was not found in LPW (*TC*_*y, mixed* *MSW* *collection*,  *carton*_ =  0.55). For the aluminum cover dismantled by customers and not found in the sample, a transfer coefficient toward mixed MSW based on Mika et al. [71] was used (*TC*_*y, mixed* *MSW* *collection*, *aluminum*_ =  0.833). Consequentially, the transfer coefficient of the dismantled aluminum cover toward separate LPW collection (F3) is *TC*_*y, LPW, aluminum*_ =  0.167. Both TC were chosen because the aforementioned studies also focused on Vienna in the year 2022.

Having determined the material flows of subcomponents for each subgood (decoration type) *y*, the sum over all decoration types ${\dot{m}}_{yi,subcomponent}$ can be built to determine the total material flows of each subcomponent ${\dot{m}}_{i,subcomponent}$, using Equation (11):

(11) $${\dot{m}}_{i,subcomponent}=\sum_{y=1}^{y=k}{\dot{m}}_{yi,subcomponent}\;$$

## 3 Results and Discussion

### 3.1 Content of Yoghurt Cups and Decoration Technologies in Analyzed Waste Streams

**FIGURE 4:**
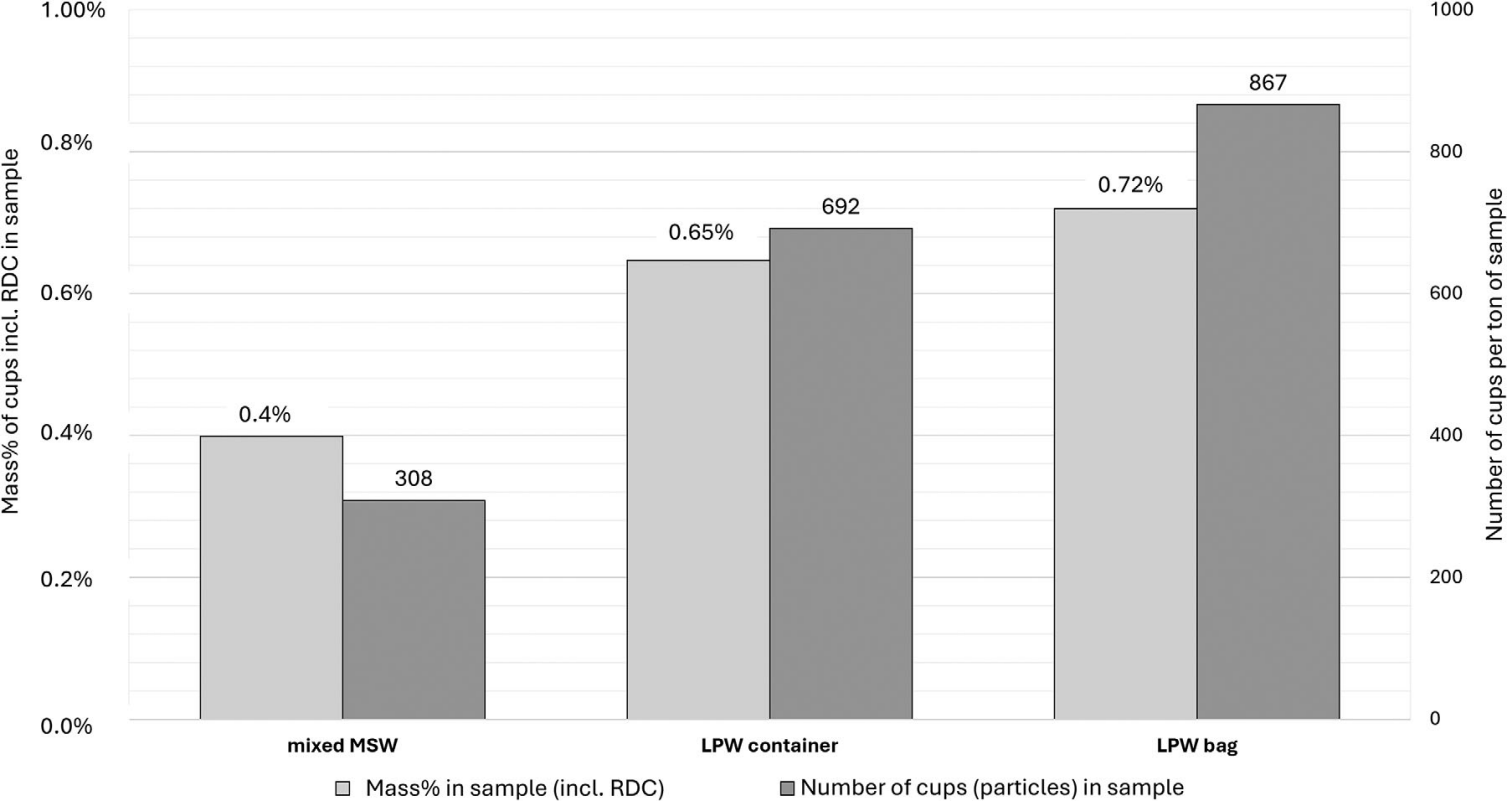
Content (in mass%) of yoghurt cups in mixed MSW, LPW containers and bag collection (including residues and dirt content‐ RDC) in sample and number of cups per ton.

In all three analyzed waste streams, the content of yoghurt cups (including RDC) is below 1 mass%, with the lowest share in mixed MSW (see Figure 4). Per ton, most cups are found in the separate LPW collection, with twice the amount of cups in LPW containers and three times the amount in LPW bags than in mixed MSW. It must be pointed out, however, that the total waste flow of mixed MSW is around forty‐five times higher than the LPW flow, as is shown in Section 2.3.1. Mass information for individual cups per waste stream is listed in Table S2.

**FIGURE 5:**
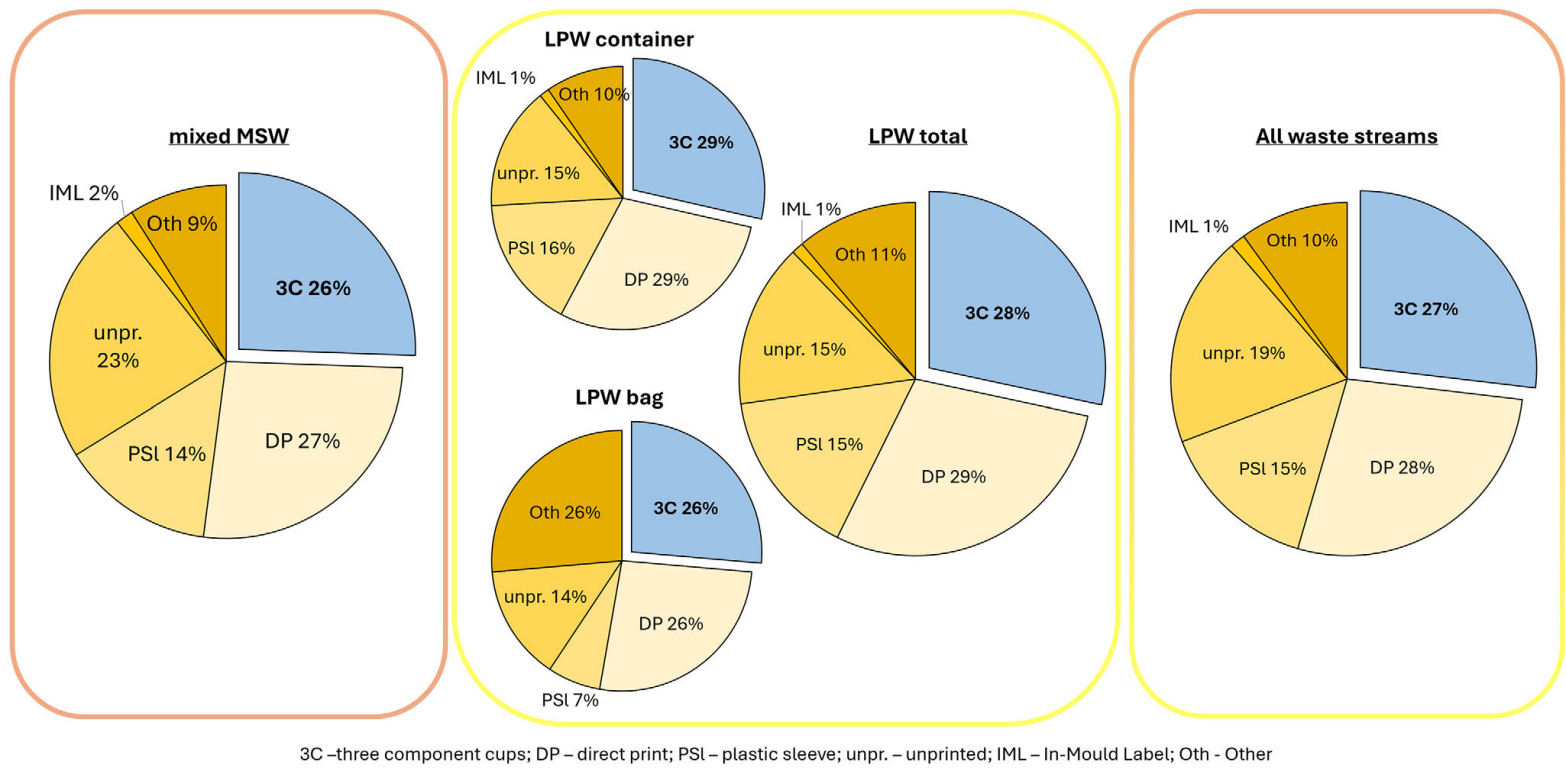
Decoration types of yoghurt cups in mixed MSW, LPW containers and LPW bags in Vienna, based on cup (particle) number. Decoration types: Direct print (DP), plastic sleeve (PSl), unprinted (unpr.), In‐Mould Label (IML), Other (Oth), yoghurt cups with carton wrap (3C). Exemplary pictures of various decoration types are listed in Figure S2. Particle numbers analyzed: 1135 in mixed MSW, 907 in LPW containers, 91 in LPW bags.

A total of six different decoration technologies were distinguished as shown in Figure 5. Across all waste streams, direct print cups (DP) are 28% of all analyzed cups by number, followed closely by 3C. The 3C content is higher in this study than in Kostic et al. [42] (18% of cups in Munich), but much lower than in Köck et al. [59] (50% of cups in a district of Vienna). The share of each decoration technology in mixed MSW and the total LPW collection is similar, with a higher share of unprinted cups (23%) in mixed MSW than in LPW (15%). While other decoration technologies, such as paper stickers, dominate in the LPW bag collection, the share of sampled cups from this waste stream was much lower than in the LPW container collection. However, LPW bags only make up a small share of the total LPW waste flow in Vienna (see Section 3.4.1).

### 3.2 Composition and Constitution of Yoghurt Cups

#### 3.2.1 Composition of Cups Including Subcomponents and Residues and Dirt Content (RDC)

**FIGURE 6:**
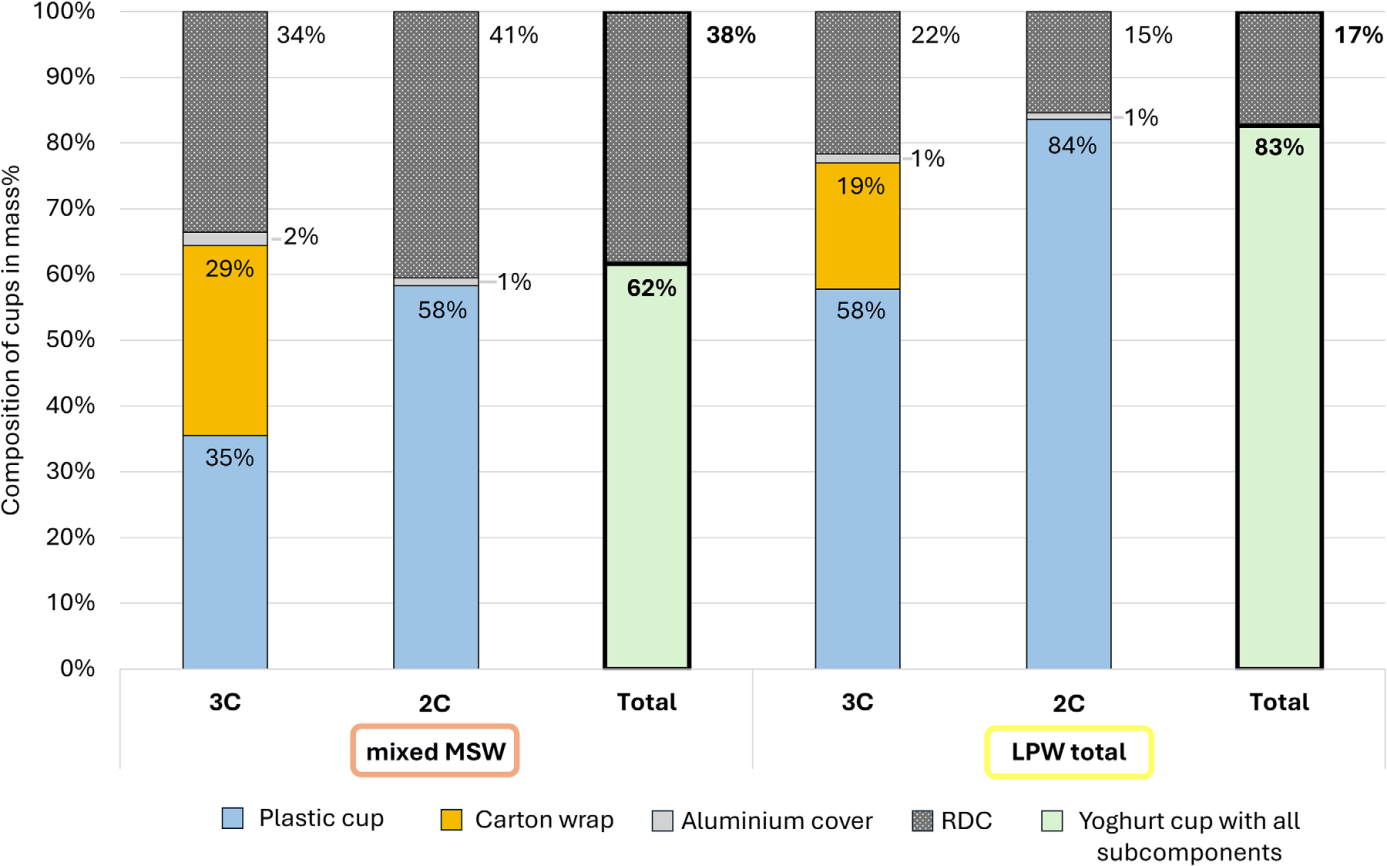
Composition of yoghurt cups in mixed MSW and total LPW collection in mass%, including residues and dirt content (RDC). Moisture content is included in the RDC. The “total” column of each waste stream indicates the share of the entire yoghurt cups, including all subcomponents, in comparison to the residues and dirt content. 3C = cups with carton wrap, 2C = cups without carton wrap.

The results of dismantling, washing, and drying of the sampled yoghurt cups are shown in Figure 6.

A larger share of RDC in cups from mixed MSW with 38% is noticeable in comparison to the share of 17% in LPW. Other studies like van Velzen et al. [30], Nemat et al. [51] or Langley et al. [72] also observed that packaging, where leftover product residue after consumption is present, is often disposed of in mixed MSW due to the consumers' perception of the packaging as “dirty” and difficult to clean. This is especially the case for yoghurt packaging, due to higher viscosity of the product [51]. When consumers, however, decide to dispose of yoghurt cups in LPW, they are more likely to clean the packaging for sanitary reasons and to reduce odor [51], which aligns with the findings in this study. The smaller mass share of plastic cups in 3C cups in comparison to other decoration types is also evident, as the additional carton wrap allows for the use of thinner plastic cups [42]. Further data regarding cup composition without RDC and distribution of cup filling volume and RDC can be found in Figures S4 and S5.

### 3.3 Constitution of Yoghurt Cups in Analyzed Waste Streams

These results only display the dismantling behavior for cups that clearly were 3C cups and 2C cups that had an aluminum cover. For data regarding cups, where no covers made from aluminum were present (e.g., plastic foil covers), see Table S3.

**FIGURE 7:**
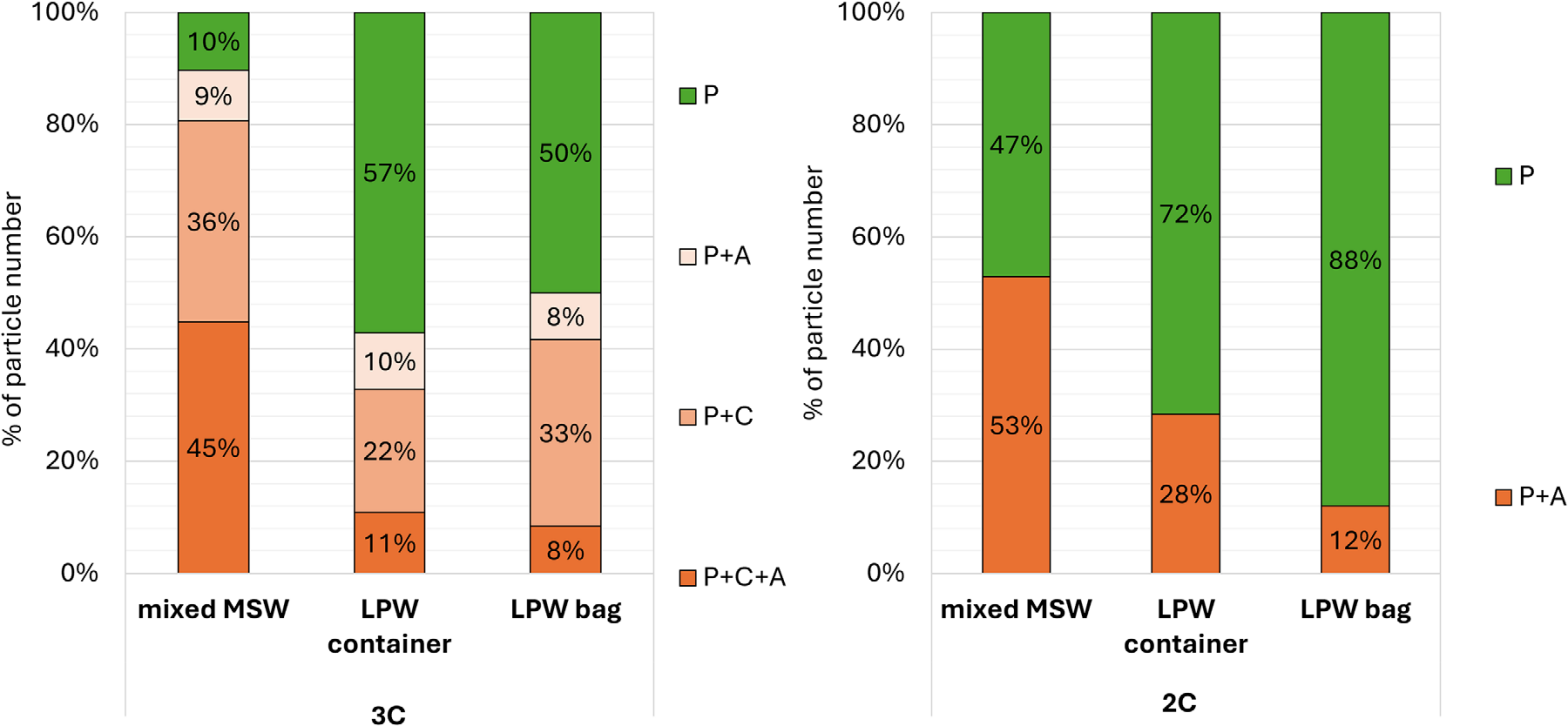
Dismantling stages of plastic yoghurt cups in analyzed waste streams, distinguishing 3C cups (with carton wrap) and other decoration technologies (2C, without carton wrap). Beneficial dismantling stages for recycling are green; undesired ones are orange. P = plastic, P+A = plastic + aluminum, P+C = plastic + carton, P+C+A = plastic + carton + aluminum. Results based on cup (particle) number.

Yoghurt cup samples were analyzed regarding their constitution to observe the dismantling and separation behavior of the consumers. In Figure 7, the found constitution per waste stream is presented, additionally distinguishing 3C cups and other decoration technologies as 2C cups. In the case of 3C cups, a significant difference in dismantling behavior can be seen between mixed MSW and LPW collection. While in both LPW streams, the share of cups that are completely dismantled (only the plastic cup remained) reaches at least 50%, around 45% of 3C cups in mixed MSW still have all their subcomponents attached. This could mean that consumers, who dispose of 3C cups in LPW, are also more likely to dismantle them, as intended. The second largest share of separately collected 3C cups, however, still has a carton wrap, which can lead to misplaced cups during the sorting process [58], even though the consumers have sorted the cups into the correct waste stream. Regarding cups with other decoration technologies, a positive sorting behavior can also be observed in LPW. 72% of cups in LPW containers and even 88% of cups in LPW bag collection are free from their aluminum covers, which further indicates a higher dismantling and separation awareness for these consumers, who participate in the separate collection of LPW.

### 3.4 Material and Particle Flows of Yoghurt Cups in Analyzed Waste Streams

This section presents material and particle flows of yoghurt cups for Vienna, 2022. The given values are rounded, while detailed values are provided in Tables S4 and S5.

#### 3.4.1 Particle Flows of Yoghurt Cups With and Without Carton Wrap

**FIGURE 8:**
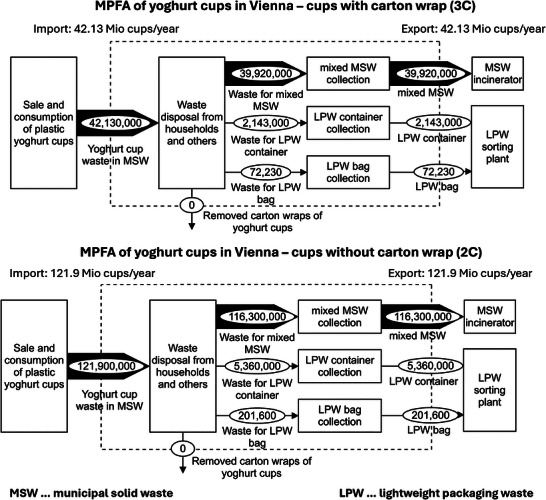
Material and particle flow analysis (MPFA) of 3C cups (top) and cups with other decoration technologies (2C) (bottom) in Vienna 2022 in cups per year. Values are rounded to 4 significant digits.

In Figure 8, the particle flows of yoghurt cups in cups per year are presented for Vienna, 2022. A distinction was made between 3C cups and 2C cups. The listed particles are equal to 645 t/yr of 3C cups and 1450 t/yr of 2C cups in Vienna. In both cases, mixed MSW is clearly portrayed as the number one waste stream for yoghurt cups, with a total of 95% of cups being discarded there, with no significant differences between the share of 3C cups and 2C cups. As mentioned in Chapter 2.3, however, the analysis was conducted before yoghurt cups became a target fraction for LPW in Vienna in 2023 [73]. A higher share of cups in LPW can therefore be expected.

The results also emphasize the difference between LPW container and bag collection. While the container collection is located at collection centers in densely populated areas, bag collection is only present in single‐family household areas at the urban fringe, amounting to only around 3% of the entire LPW [16]. An MFA of all yoghurt cups on a wet matter basis can be found in Figure S6.

#### 3.4.2 Material Flow of Yoghurt Cup Subcomponents in Analyzed Waste Streams

**FIGURE 9:**
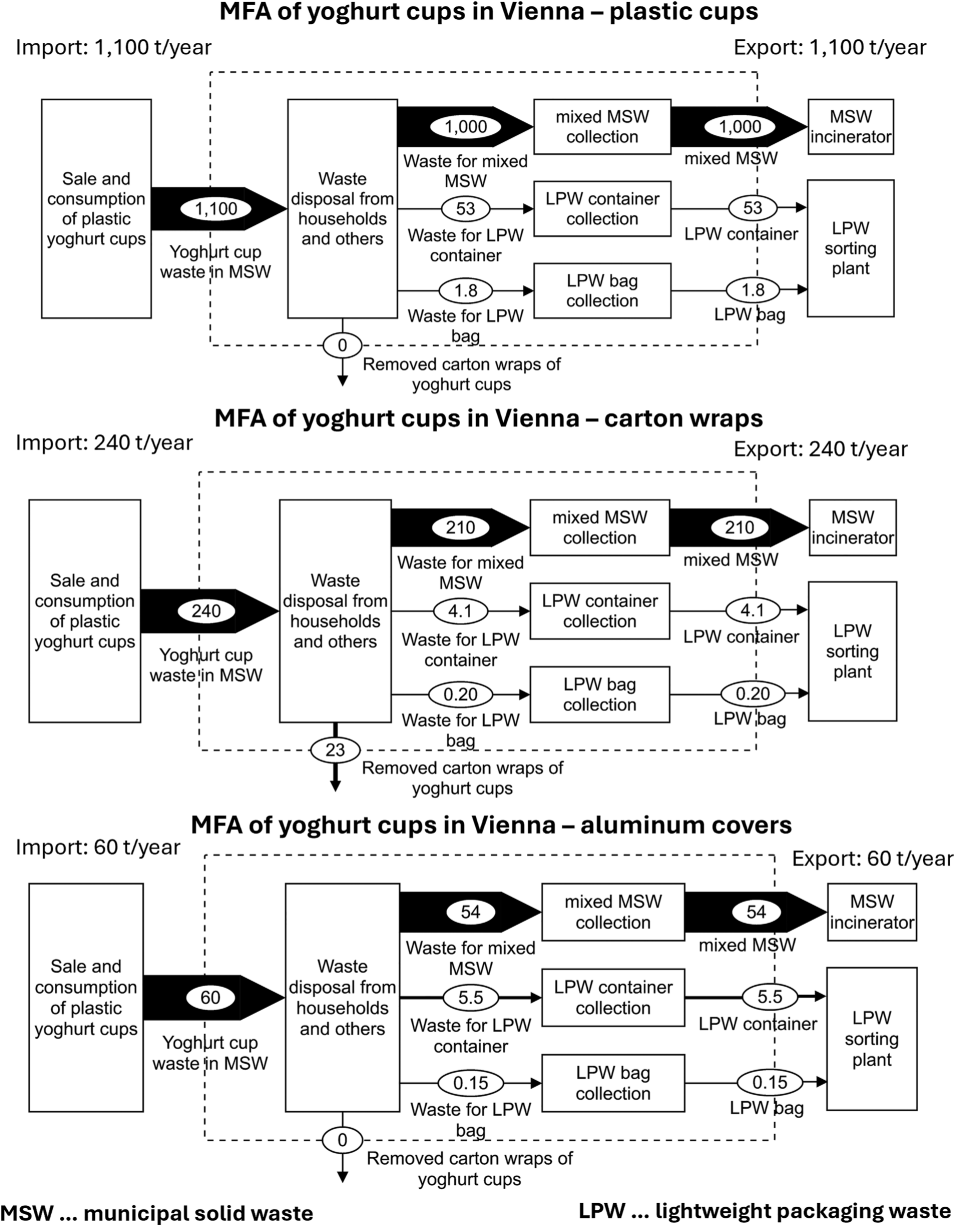
Material flow analysis of yoghurt cup subcomponents (plastic cups, carton wraps, aluminum covers) for Vienna, 2022 in tons/year on a dry matter basis. Values are rounded to two significant digits.

The material flows of yoghurt cup subcomponents (plastic cup, carton wrap, aluminum cover) for Vienna 2022 are presented in Figure 9 in tons per year. Every type of subcomponent is primarily found in mixed MSW, with plastic cups showing the largest share of around 1000 t/yr. These cups, which primarily consist of polypropylene and polystyrene (see Table S1), end up in the MSW incinerator and are lost for mechanical recycling. Only 5% of plastic cups (dry matter basis) are collected separately over the LPW container and bag collection. While disposal of plastic yoghurt cups over other waste streams than mixed MSW and LPW collection cannot be ruled out, their share can be assumed minimal as past sorting analyses have determined mixed MSW and LPW collection as the only relevant waste streams containing yoghurt cups (see Chapter 2.1.1). Other analyzed waste streams during the aforementioned sorting analysis, such as waste paper, glass, organics or waste collected from public spaces, all showed a content of <1 mass% of non‐targeted recyclable material (e.g., plastics found in waste paper), which means that other waste streams are only marginal sinks for yoghurt cups [62].

Regarding the carton wraps of 3C yoghurt cups, around 88% are found in mixed MSW while around 10% are removed from the cups and are ideally disposed of via wastepaper collection, as intended. Further research, however, is needed to determine whether carton wraps of 3C cups land in this waste stream. Only about 2% of carton wraps find their way into the separate LPW collection, which is beneficial due to the aforementioned difficulty of accurate polymer recognition [57, 58], as well as the varying degrees of recyclability of carton‐wrapped cups depending on the region [41]. However, a new rendition of the 3C cup promises a self‐separating carton wrap that detaches from the cup during the sorting of LPW [74]. While this can decrease the share of missorted cups, it hinders high‐quality recycling of the carton wraps, as paper from mixed waste streams such as LPW shows lower qualities [75, 76] than separately collected wastepaper. In Germany, the German Federal Institute for Risk Assessment additionally forbids the reuse of paper from mixed waste streams and its use for food packaging [77], which in total lowers the recyclability of the 3C cup. Aluminum covers make up the smallest share of all analyzed yoghurt cup subcomponents with a total of 60 t/yr, with 90% of them landing in mixed MSW. While aluminum can be recovered after the incineration process, recovery rates of aluminum from bottom ash originating from foils are very low, which is due to fragmentation and oxidization of the thin metal [78, 79].

### 3.5 Toward a Circular Economy of Yoghurt Cups

Despite plastic yoghurt cups being the subject of discussion in both the public and science, surprisingly little data on this packaging, particularly the different production and decoration types and how and where they are disposed of in waste, is available. With this study, some research gaps were filled, demonstrating that design‐for‐recycling efforts can be compromised by lacking participation of consumers, for instance when it comes to dismantling and correctly disposing of the packaging components to separate collection schemes.

However, the study at hand also suggests that still more data is needed so that both policy makers and packaging companies make informed decisions on packaging and design‐for‐recycling. First, the study was conducted in Vienna, and of course, future research should focus on larger geographical entities, like Austria, due to the differences in the separate collection rate of plastic packaging waste among the different federal states in the country. While rural areas tend to achieve higher separate collection rates [80], it is unclear whether a higher degree of dismantling of yoghurt cup subcomponents can be expected as well. Moreover, the popularity of 3C yoghurt cups in the DACH region suggests that similar studies should be carried out for Germany and Switzerland. The present work provides a sound methodological base for studies like these.

**FIGURE 10:**
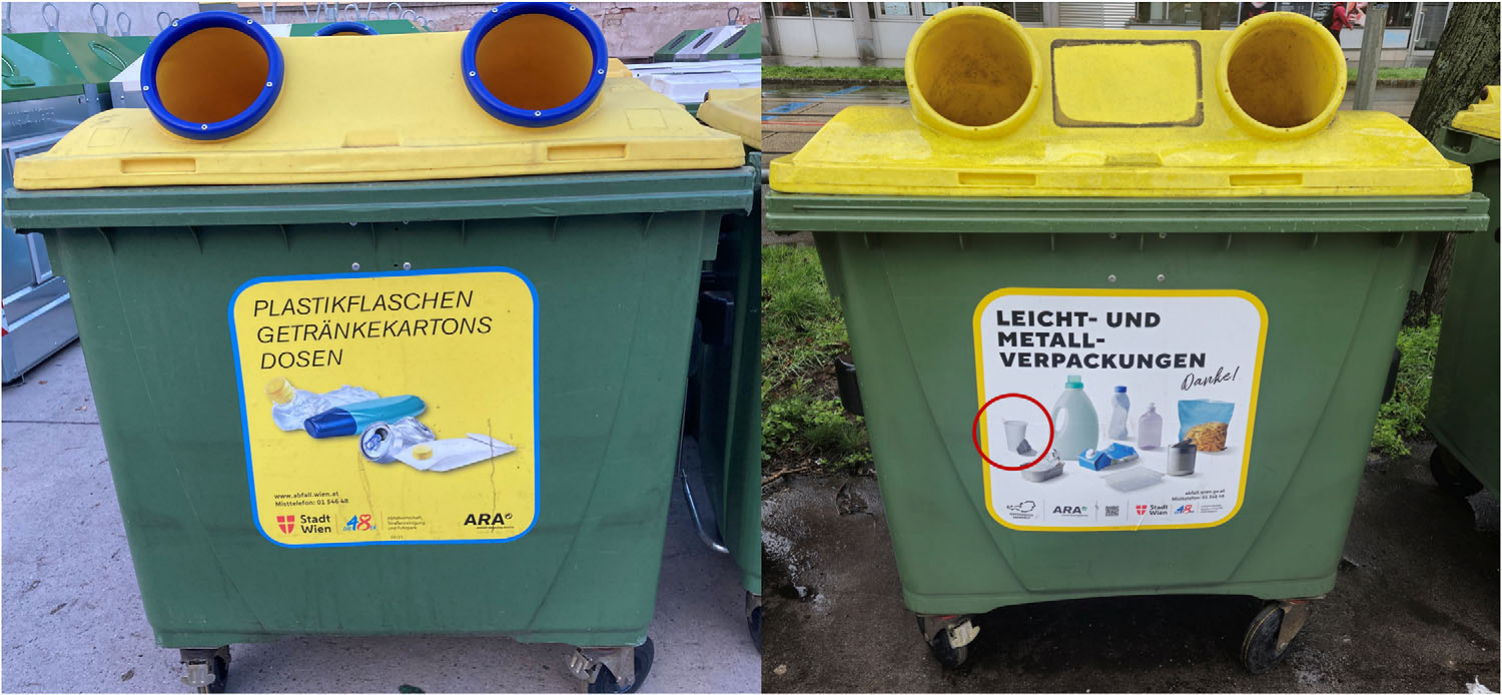
Lightweight packaging waste (LPW) collection containers in Vienna with old (left) and new pictograms (right). Contrary to the old pictogram, the new one promotes the separate collection of completely dismantled yoghurt cups (highlighted in red). Pictures by Lea Gritsch and Gisela Breslmayer.

Furthermore, at the time of the sampling campaign in Vienna in the year 2022, yoghurt cups were not actively promoted for separate collection, as can be seen in Figure 10. Since the year 2023, however, it was made mandatory by law that all plastic packaging, including yoghurt cups, must be promoted for separate LPW collection in Austria [73]. Therefore, Vienna has introduced new pictograms on its LPW containers in the year 2025, explicitly promoting a fully dismantled yoghurt cup, along with other lightweight packaging waste. This new pictogram is shown in Figure 10 in comparison to the previous version. Based on that, more separately collected and better dismantled yoghurt cups could be disposed of into separate LPW collection. However, since these new containers were only introduced one year ago, it was hitherto not yet possible to evaluate whether the separate collection and dismantling behavior of customers has been influenced. Updated data can, however, be expected in the future, due to currently conducted and planned sorting analysis to supervise the changed sorting behavior of consumers. Overall, a large potential is left regarding consumer education to encourage separate collection of packaging waste.

Better monitoring of the content and quality of plastic packaging like yoghurt cups in different waste streams is necessary, for instance by conducting similar sampling campaigns as the one presented in this study. Hereby, it should be determined where dismantled subcomponents like carton wraps and aluminum covers are disposed of by customers, as this study partly based the data on existing studies conducted in Vienna. A big challenge here is the little particle number of these subcomponents would require an increase in the sampled mass. This way, data to better plan a design‐for‐recycling‐based circular economy of yoghurt cups would be available.

## 4 Conclusion

Yoghurt cups from mixed municipal solid waste and separate lightweight packaging waste collection were obtained in Vienna, 2022, and analyzed according to decoration and polymer type, residues and dirt content, and dismantling stage—meaning to what degree consumers separate yoghurt cups into their subcomponents. Furthermore, material flows of yoghurt cups and their subcomponents were modelled on a particle and mass basis for the analyzed waste streams. Special focus was put on three‐component (3C) yoghurt cups, consisting of a plastic cup, an aluminum cover, and a carton wrap, which should be removed by the consumers before disposal. This decoration type was one of the most used ones for yoghurt cups, as the results from the conducted hand sorting analysis showed. Looking at the dismantling stage of separately collected cups, between 50% and 57% of 3C cups and 72% and 88% of yoghurt cups with other decoration types were completely separated from their subcomponents, therefore enabling recycling of the plastic cup. However, 95% of all yoghurt cups (by number) in Vienna were disposed of via mixed municipal solid waste and further incinerated, resulting in about 164 million yoghurt cups, primarily consisting of polypropylene, which are lost for mechanical recycling.

While the individual subcomponents of 3C cups are recyclable, the findings emphasize the lack of participation when it comes to dismantling of the cups, especially considering the carton wrap. This can lead to missorted cups in the sorting facility while generating a low‐quality carton waste stream. With their increasing usage in the DACH region and with Germany being the largest yoghurt producer in the EU‐27, it is therefore recommended to reconsider these types of yoghurt cups with attention to design‐for‐recycling. Further waste sampling analyses of such packaging, especially in the DACH region, are recommended to improve monitoring of this waste stream. Avoiding multi‐material packaging and the consumer‐dependent separation thereof additionally enables the circularity of such packaging in the EU.

## Funding

This work was financially supported by the Austrian Federal Ministry for Economy, Energy and Tourism, the National Foundation for Research, Technology and Development and the Christian Doppler Research Association is gratefully acknowledged. The authors also acknowledge TU Wien Bibliothek for financial support through its Open Access Funding Program.

## Conflicts of Interest

The authors declare no conflicts of interest.

## Supporting information




**Supporting File**: gch270144‐sup‐0001‐SuppMat.docx.

## Data Availability

The data that supports the findings of this study are available in the supplementary material of this article.
