# Correction to “Characteristics and Material Flows of Diary Packaging in Municipal Solid Waste: A Case Study of Plastic Yoghurt Cups in Vienna”

**DOI:** 10.1002/gch2.70169

**Published:** 2026-10-18

G. Breslmayer, L. Gritsch, and J. Lederer, “Characteristics and Material Flows of Diary Packaging in Municipal Solid Waste: A Case Study of Plastic Yoghurt Cups in Vienna,” *Global Challenges* 10, no. 8 (2026): e70144, https://doi.org/10.1002/gch2.70144.

In the title of this paper, the use of the term “Diary” was incorrect. The correct term instead is “Dairy.”

We apologize for this error.

