# Supplementary material for: Characteristics and Material Flows of Diary Packaging in Municipal Solid Waste: A Case Study of Plastic Yoghurt Cups in Vienna
Source: Glob Chall. 2026 Aug 12;10(8):e70144. doi: 10.1002/gch2.70144 (PMC13468905; doi:10.1002/gch2.70144)
Supplement: Supplementary file 1 [file GCH2-10-e70144-s001.docx]

Characteristics and material flows of diary packaging in municipal solid waste: a case study of plastic yoghurt cups in Vienna

Supplementary-file

Gisela Breslmayer^*^, Lea Gritsch, Jakob Lederer

Christian Doppler Laboratory for a recycling-based Circular Economy, Institute of Chemical, Environmental and Bioscience Engineering, Technische Universität Wien, Getreidemarkt 9, 1060 Vienna, Austria

* Corresponding author: Gisela Breslmayer, gisela.breslmayer @tuwien.ac.at

Table of contents

[Abbreviations	2](#_Toc234762519)

[S1.	Introduction	3](#_Toc234762520)

[S2.	Materials and methods	3](#_Toc234762521)

[S2.1	Waste sampling and analysis of the yoghurt cup content and composition	3](#_Toc234762522)

[S2.1.1	Collection and analysis of primary waste samples to determine the yoghurt cup content	3](#_Toc234762523)

[S2.1.2	Sorting of yoghurt cups according to their decoration type	3](#_Toc234762524)

[S2.2	Constitution, subcomponents, residues and dirt content of yoghurt cups in samples	4](#_Toc234762525)

[S2.2.1	Constitution (dismantling stage)	4](#_Toc234762526)

[S2.2.2	Analysis of subcomponents	4](#_Toc234762527)

[S2.2.3	Residues and dirt content (RDC)	4](#_Toc234762528)

[S2.3	Modelling material and particle flows of yoghurt cups in Vienna	5](#_Toc234762529)

[S2.3.1	Material flows of goods	5](#_Toc234762530)

[S2.3.2	Particle flows of subgoods	5](#_Toc234762531)

[S2.3.3	Material flows of subcomponents	5](#_Toc234762532)

[S3.	Results and discussion	5](#_Toc234762533)

[S3.1	Content of yoghurt cups and decoration technologies in analyzed waste streams	5](#_Toc234762534)

[S3.2	Composition and constitution of yoghurt cups	6](#_Toc234762535)

[*S3.2.1*	Composition of cups including subcomponents and residues and dirt content (RDC)	6](#_Toc234762536)

[S3.3	Constitution of yoghurt cups in analyzed waste streams	7](#_Toc234762537)

[S.3.4 Material and particle flows of yoghurt cups in analyzed waste streams	8](#_Toc234762538)

[*S.3.4.1 Particle flows of yoghurt cups with and without carton wrap*	8](#_Toc234762539)

[*S3.4.2. Material flow of yoghurt cup subcomponents in analysed waste streams*	9](#_Toc234762540)

[S3.5 Towards a circular economy of plastic yoghurt cups	9](#_Toc234762541)

[S4.	Conclusion	9](#_Toc234762542)

# Abbreviations

3C – 3 component yoghurt cup with carton wrap

DP – direct print

IML – In-Mould Labelling

LPW – Lightweight packaging waste

MPFA – Material and particle flow analysis

MSW – Municipal solid waste

Oth – Other cup decoration types

PET – Polyethylenterephthalate

PP– Polypropylene

PS – Polystyrene

PSl – Plastic sleeve

RDC – residues and dirt content

Unpr. – unprinted

# S1.  Introduction

Figure S 1: Separation and disposal instructions found on 3 component yoghurt cups. (From left to right) First cup: Usage of pictograms to promote the separate disposal of plastic cup, aluminum cover and carton wrap; Second cup: “Tear open here to recycle”; Third cup: “Open here and separate for recycling” with an image indicating the different materials of the cup; Fourth cup: “Please peel off here and dispose of separately.” with the additional incentive of a lottery on the inside of the carton wrap.

# S2.  Materials and methods

## S2.1 Waste sampling and analysis of the yoghurt cup content and composition

### S2.1.1 Collection and analysis of primary waste samples to determine the yoghurt cup content

### S2.1.2 Sorting of yoghurt cups according to their decoration type

Figure S 2: Decoration types of yoghurt cups. Top row from left to right: direct print, in-mould label, plastic sleeve. Bottom row from left to right: Other, unprinted,
3 component cups.

#### Table S1: Decoration and polymer types of yoghurt cups on a dry matter basis

| **Waste stream Decoration type** | **LPW bag (kg)** | **LPW container (kg)** | **mixed MSW (kg)** | **Total (kg)** |
| --- | --- | --- | --- | --- |
| **Direct print (DP)** | **0,23** | **1,99** | **2,36** | **4,58** |
| PET | 0,01 | 0,06 | 0,09 | 0,16 |
| PP | 0,15 | 1,08 | 1,23 | 2,46 |
| PS | 0,06 | 0,85 | 1,04 | 1,95 |
| Other polymers |  |  | 0,01 | 0,01 |
| **In-Mould Labelling (IML)** |  | **0,16** | **0,29** | **0,44** |
| PP |  | 0,16 | 0,29 | 0,44 |
| **3C** | **0,19** | **2,09** | **3,00** | **5,29** |
| PP | 0,16 | 1,72 | 2,23 | 4,10 |
| PS | 0,04 | 0,37 | 0,76 | 1,17 |
| Other polymers |  |  | 0,02 | 0,02 |
| **Other** | **0,12** | **0,55** | **0,68** | **1,35** |
| PET |  |  | 0,00 | 0,00 |
| PP |  | 0,16 | 0,19 | 0,34 |
| PS | 0,12 | 0,40 | 0,49 | 1,00 |
| **PSl** | **0,04** | **1,20** | **1,45** | **2,70** |
| PP | 0,04 | 1,20 | 1,43 | 2,68 |
| PS |  |  | 0,02 | 0,02 |
| **Unpr.** | **0,10** | **0,95** | **1,28** | **2,33** |
| PET |  | 0,41 | 0,02 | 0,43 |
| PP | 0,09 | 0,22 | 0,44 | 0,75 |
| PS | 0,01 | 0,29 | 0,77 | 1,07 |
| Other polymers |  | 0,03 | 0,05 | 0,07 |
| **Total (kg)** | **0,68** | **6,95** | **9,06** | **16,69** |

## S2.2 Constitution, subcomponents, residues and dirt content of yoghurt cups in samples

### S2.2.1 Constitution (dismantling stage)

### S2.2.2 Analysis of subcomponents

### S2.2.3 Residues and dirt content (RDC)

## S2.3 Modelling material and particle flows of yoghurt cups in Vienna

Figure S 3: Collection schemes of lightweight packaging waste (LPW), mixed municipal solid waste (MSW) and waste paper in Vienna.

### S2.3.1  Material flows of goods

### S2.3.2  Particle flows of subgoods

### S2.3.3  Material flows of subcomponents

# S3. Results and discussion

## S3.1 Content of yoghurt cups and decoration technologies in analyzed waste streams

Table S2: Mass of individual cups per waste stream (wet matter basis).

| **Mixed MSW (kg)** | **LPW container (kg)** | **LPW bag (kg)** | **Total (kg)** |
| --- | --- | --- | --- |
| 0.013 | 0.009 | 0.008 | 0.013 |

## S3.2 Composition and constitution of yoghurt cups

### S3.2.1 Composition of cups including subcomponents and residues and dirt content (RDC)

*Figure S 4* *Composition of yoghurt cups without residues and dirt content (RDC) (dry matter basis)*

Figure S 5: Residues and dirt content (RDC) per waste stream, decoration type and filling volume in grams, with number of cups given per filling volume, waste stream and decoration type.

## S3.3 Constitution of yoghurt cups in analyzed waste streams

Table S3: Number of cups per waste stream and decoration type with removed and present aluminum covers and no aluminum covers (e.g. plastic foil covers).

| **Decoration type** | **Waste stream** | **alu-cover removed** | **with alu-cover** | **no alu-cover** |
| --- | --- | --- | --- | --- |
| **3C** | mixed MSW | 134 | 156 |  |
|  | LPW container | 205 | 54 |  |
|  | LPW bag | 20 | 4 |  |
| **DP** | mixed MSW | 136 | 154 | 11 |
|  | LPW container | 181 | 72 | 12 |
|  | LPW bag | 16 | 3 | 5 |
| **PSl** | mixed MSW | 82 | 77 | 1 |
|  | LPW container | 112 | 36 |  |
|  | LPW bag | 6 |  |  |
| **unpr.** | mixed MSW | 3 | 6 | 255 |
|  | LPW container | 1 | 4 | 132 |
|  | LPW bag |  |  | 13 |
| **IML** | mixed MSW | 3 | 8 | 7 |
|  | LPW container | 3 | 6 | 2 |
|  | LPW bag |  |  |  |
| **Oth** | mixed MSW | 6 | 14 | 82 |
|  | LPW container | 10 | 4 | 73 |
|  | LPW bag |  |  | 24 |

## S.3.4 Material and particle flows of yoghurt cups in analyzed waste streams

### *S.3.4.1 Particle flows of yoghurt cups with and without carton wrap*

Figure S 6: Material flow of all yoghurt cups, 3C and 2C cups in Vienna, 2022 on a wet-matter basis, rounded to 3 significant digits.

Values for Table S3 are listed according to the Material flow analysis model in Figure 2 with F1 to F8.

Table S4: Detailed values (number of cups) for MPFA of yoghurt cups in Vienna, 2022 with carton wrap (3C) and without carton wrap (2C).

| **3C Cups** | **F1** | **F2** | **F3** | **F4** | **F6** | **F7** | **F8** |
| --- | --- | --- | --- | --- | --- | --- | --- |
|  | 42,134,073 | 39,919,315 | 2,142,529 | 72,229 | 39,919,315 | 2,142,529 | 72,229 |
| **2C Cups** | 121,878,722 | 116,316,626 | 5,360,458 | 201,638 | 116,316,626 | 5,360,458 | 201,638 |

### *S3.4.2. Material flow of yoghurt cup subcomponents in analysed waste streams*

Values for Table S4 are listed according to the Material flow analysis model in Figure 2 with F1 to F8.

Table S5: Detailed values in t/yr for MFA of yoghurt cup subcomponents (plastic cups, carton wraps, aluminum covers) in Vienna, 2022.

| **Plastic cups** | **F1** | **F2** | **F3** | **F4** | **F5** | **F6** | **F7** | **F8** |
| --- | --- | --- | --- | --- | --- | --- | --- | --- |
|  | 1,069.47 | 1,015.18 | 52.45 | 1.84 | 0 | 1,015.18 | 52.45 | 1.84 |
| **Carton wraps** | 236.25 | 208.51 | 4.09 | 0.20 | 23.46 | 208.51 | 4.09 | 0.20 |
| **Alu-covers** | 59.87 | 54.18 | 5.55 | 0.15 | 0 | 54.18 | 5.55 | 0.15 |

## S3.5 Towards a circular economy of plastic yoghurt cups

N.D.

# S4. Conclusion

N.D.
